# In Vitro Cytotoxicity of D18 and Y6 as Potential Organic Photovoltaic Materials for Retinal Prostheses

**DOI:** 10.3390/ijms23158666

**Published:** 2022-08-04

**Authors:** Ana Cetkovic, Alessandro Bellapianta, Mihai Irimia-Vladu, Jakob Hofinger, Cigdem Yumusak, Andrea Corna, Markus Clark Scharber, Günther Zeck, Niyazi Serdar Sariciftci, Matthias Bolz, Ahmad Salti

**Affiliations:** 1 Center for Medical Research, University Clinic for Ophthalmology and Optometry, Johannes Kepler University Linz, 4020 Linz, Austria; 2 Linz Institute of Organic Solar Cells (LIOS), Institute of Physical Chemistry, Johannes Kepler University Linz, Altenbergerstrasse 69, 4040 Linz, Austria; 3 Institute of Biomedical Electronics, Technische Universität (TU) Wien, 1040 Vienna, Austria

**Keywords:** retinal dystrophy, cytotoxicity, retinal prosthesis, D18, Y6, organic photovoltaic materials

## Abstract

Millions of people worldwide are diagnosed with retinal dystrophies such as retinitis pigmentosa and age-related macular degeneration. A retinal prosthesis using organic photovoltaic (OPV) semiconductors is a promising therapeutic device to restore vision to patients at the late onset of the disease. However, an appropriate cytotoxicity approach has to be employed on the OPV materials before using them as retinal implants. In this study, we followed ISO standards to assess the cytotoxicity of D18, Y6, PFN-Br and PDIN individually, and as mixtures of D18/Y6, D18/Y6/PFN-Br and D18/Y6/PDIN. These materials were proven for their high performance as organic solar cells. Human RPE cells were put in direct and indirect contact with these materials to analyze their cytotoxicity by the MTT assay, apoptosis by flow cytometry, and measurements of cell morphology and proliferation by immunofluorescence. We also assessed electrophysiological recordings on mouse retinal explants via microelectrode arrays (MEAs) coated with D18/Y6. In contrast to PFN-Br and PDIN, all in vitro experiments show no cytotoxicity of D18 and Y6 alone or as a D18/Y6 mixture. We conclude that D18/Y6 is safe to be subsequently investigated as a retinal prosthesis.

## 1. Introduction

Vision is one of if not the most valuable of the senses, allowing us to discover the world we live in. The human retina is a highly complex organ that represents an important part of the central nervous system. It is often called the window to the brain, and dysfunction of retinal neurons is a major cause of incurable blindness worldwide. Millions of people are being diagnosed with retinal dystrophies, ranging from inherited retinal degenerations such as retinitis pigmentosa, to age-related diseases such as age-related macular degeneration. The extent of symptoms greatly varies starting from night or colour blindness and extending to tunnel vision and complete blindness [1]. Despite the advances in understanding retinal development, to date, no treatments or definitive cures are available to reverse the degenerative processes of retinal dystrophies or to restore vision with blindness. Different therapeutic approaches are employed at early stages of the disease progression, such as gene replacement therapy and pharmacological treatment [2,3]. During the later stages of retinal degeneration, where many photoreceptors are lost, optogenetic therapy, stem cell therapy and retinal prostheses are promising strategies to restore vision in retinal dystrophy patients [2,4].

The basic principle of retinal prostheses is to recover the visual function by an adequate replacement of damaged or lost photoreceptors in the degenerated retina [5]. This is achieved successfully when the loss of retinal cells is mostly restricted to the outer layer of the retina, while the neurons in the middle and inner layers remain nearly intact [6,7]. To achieve artificial retina stimulation, distinct approaches are pursued: electrical stimulation based on external goggle information [8,9] and electrical stimulation based on implanted photovoltaic electronics [10,11]. While the former relies on directing a stimulating current to the electrodes in or near the retina, the latter utilizes the incident light to generate electrical stimulation via micro-photodiode arrays (MPDA) [11,12]. Devices that were commercially available, such as Argus II System and Alpha-AMS/IMS [8], were based on the classic electronics and inorganic materials but their manufacture was subsequently suspended. Currently, the silicon-based PRIMA implant is another device being tested in clinical trials [9]. Nevertheless, organic semiconductors have gained great attention in recent years as potential materials for organic electronic devices owing to their photovoltaic properties, transparency, mechanical flexibility, conformability and presumed biocompatibility [4,13,14,15]. 

Organic electronics represent one of the most appealing approaches which already has demonstrated promising results in terms of neuronal stimulation, and opens an entirely new horizon for the utilization of these materials in the fabrication of retinal prostheses [4,16,17]. A pool of different organic semiconducting polymers and pigments have been investigated for their performance as organic retinal prostheses. PEDOT:PSS (poly (3,4 ethylenedioxythiophene)-poly (styrenesulfonate)) is one of the most studied materials within this field and its use for retinal stimulation has been extensively studied [18,19,20,21]. Another organic polymer, regioregular poly (3-hexylthiophene-2,5-diyl) or P3HT has received a lot of attention due to its improved efficiency and functionality when combined with PEDOT:PSS [22,23,24]. Another, even more advanced combination of materials used for successful neuronal stimulation was achieved with P3HT and [6,6]-phenyl-C61-butyric acid methyl ester (PCBM) [22,25,26]. Most of these studies focused on the materials’ performance but their safety and cytotoxicity as medical devices were not effectively addressed.

Here, we explored the cytotoxicity aspects of the organic photovoltaic (OPV) semiconductors D18 and Y6 that we previously investigated for their high performance in organic solar cells [27]. We aimed to use these organic semiconductors as donor and acceptor materials for a photovoltaic retinal implant. However, before we went through investigating this device, it was initially important to address the cytotoxicity of these OPV materials alone and in combination with other organic materials usually used as electron transport layers (ETLs) to improve the efficiency of the solar cells, such as PFN and PDIN. Therefore, we followed ISO10993-5 for the biological evaluation of medical devices [28] and investigated the cytotoxicity of both the materials and their extracts by an MTT assay, apoptosis analysis by flow cytometry, the cells’ morphology and proliferation by immunofluorescence, and the functional assessment on microelectrode arrays (MEAs). Even if the retinal pigment epithelium (RPE) itself seems to be involved in pathophysiologic processes in retinitis pigmentosa, still, the subretinal space seems to be relevant for positioning possible new photovoltaic implants [29]. Therefore, the in vitro culture experiments were conducted using the human RPE cell line, and the electrophysiological recordings were performed on ex-vivo mouse retina. 

## 2. Results

### 2.1. Selection of the Organic Semiconductor Materials

**Figure 1:**
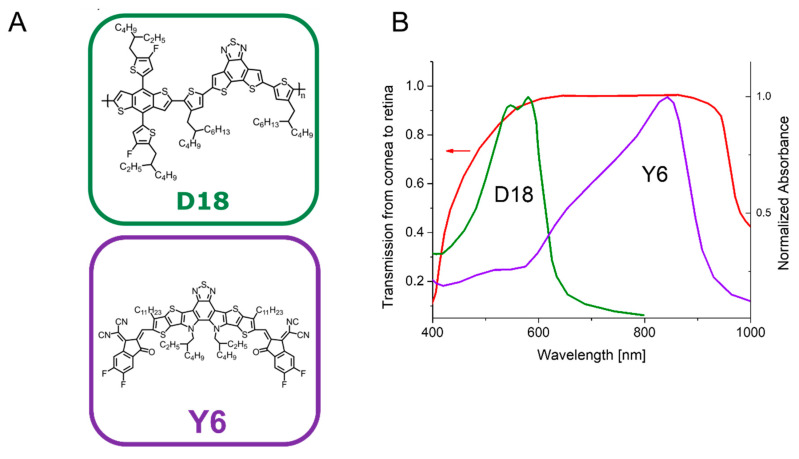
(**A**) Chemical structure of D18 and Y6; (**B**) Comparison transmission from the corona to the retina of a human eye and absorbance of D18 and Y6.

From a broad range of organic semiconductors available, we decided to select D18 and Y6 based on their high performance as solar cells [27,30] and their excellent spectral overlap with the transmission of the human eye (Figure 1). The chemical structures of D18 and Y6 are shown in Figure 1A. In Figure 1B, the transmission from the cornea to the retina of a human eye is compared to the absorbance of D18 and Y6. The chemical structures of PFN-Br and PDIN are shown in Supplementary Figure S1.

### 2.2. Cytotoxicity of the Materials’ Extract

**Figure 2:**
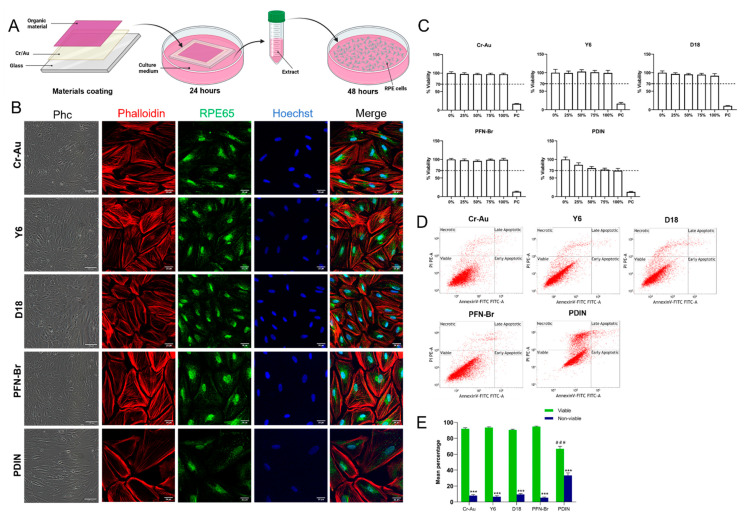
Cytotoxicity of Y6, D18, PFN-Br and PDIN extracts. (**A**) Schematic representation of the indirect contact procedure to obtain the materials’ extracts (created with Biorender.com). (**B**) RPE-19 cell line cultured with the material extracts for 48 h, shown as phase contrast and immunofluorescence images stained against the filament actin marker Phalloidin, the RPE cell marker RPE65 and the nuclei marker Hoechst. (**C**) Percentage of RPE cell viability investigated by MTT assay after 48 h of culture with each material extract at different concentrations (*n* = 3). The 70% dotted line represents the minimum viability threshold of a safe material according to ISO standards. (**D**) Representative flow cytometry plots from each material extract after staining with the apoptotic marker annexin V and the necrotic marker propidium iodine (PI). (**E**) Mean percentage of the number of viable and non viable cells (apoptotic and necrotic) from 3 independent flow cytometry experiments on RPE cells cultured with the extracts. *** *p* < 0.001 as compared to viable; ### *p* < 0.001 as compared to viable Cr–Au. Scale bar is 100 µm for the Phc images and 25 µm for the fluorescent images. RPE, retinal pigment epithelium; Phc, phase contrast; PC, positive control; PI, propidium iodine.

According to ISO 10993-5 for the biological evaluation of medical devices [28], in vitro cytotoxicity shall be performed on an extract of the test sample and on the test sample itself. Extracting conditions should determine the potential toxicological hazard without causing significant changes in the test sample, such as any alteration of the chemical structure. Therefore, we first evaluated the cytotoxicity of each material individually, namely Y6, D18, PFN-Br, and PDIN, as well as uncoated glass and chromium–gold (Cr-Au) as controls. The organic materials were coated on a glass coverslip using either spin coating or physical vapor deposition methods. All glass slides were initially coated with Cr–Au (2 nm Cr and 3 nm Au in a stacked bilayer) to enhance the attachment and stability of the materials on the glass substrate. To obtain the extracts, the coated slides were incubated for 24 h in culture medium following ISO 10993-12 [31]. The extract obtained from each material was then used as culture medium on human RPE cells with equal seeding density (Figure 2A). 

After 48 h in culture with the materials’ extract, the cells cultured with D18, Y6 and PFN-Br extracts appeared to have similar cell confluency, survival and cell morphology when compared to the Cr–Au control as shown by phase contrast images (Figure 2B). Immunofluorescence using antibodies against the RPE marker RPE65, the filament actin marker Phalloidin, and the nuclei staining Hoechst show typical RPE cell morphology when compared to controls (Figure 2B). However, the cells cultured with PDIN extract show a lower cell number, with flattened and elongated cell morphology (Figure 2B). We then evaluated the cytotoxicity of each material extract at five different dilutions by the MTT assay (Figure 2C). The graphs summarize the mean viability percentage of the cells cultured for 48 h with 0 (control), 25, 50, 75, and 100% material extract and 0.05% Triton as a positive cell death control. The results show more than 90% viability in all concentration conditions of Cr–Au, D18, Y6 and PFN extract mediums with no difference as compared to the 0% control. In contrast, a gradual decrease in cell viability that correlated with the increase of extract concentration was observed for PDIN. At 100% PDIN extract concentration, cell viability was below 70%, which is the minimum threshold for material cytotoxicity according to ISO 10993-5 (Figure 2C). To note, no difference between uncoated and Cr–Au coated glass coverslips was observed with regard to cell morphology and apoptosis (Supplementary Figure S2), so we decided to use Cr–Au as our suitable control. 

**Figure 3:**
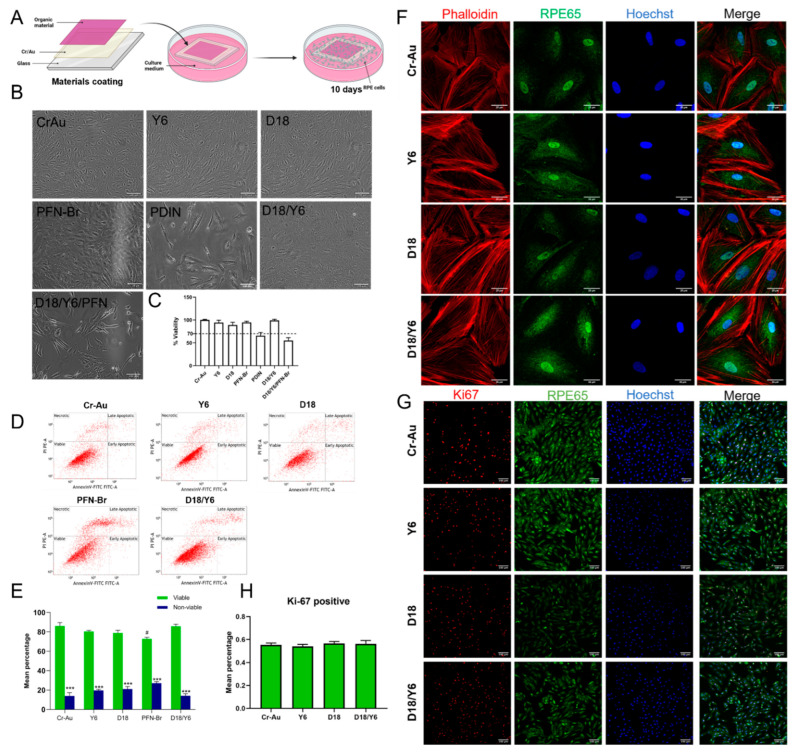
Cytotoxicity of Y6, D18, PFN-Br, PDIN, D18/Y6 and D18/Y6/PFN-Br by direct contract with human RPE cells. (**A**) Schematic representation of the direct contact procedure (created with Biorender.com). (**B**) RPE-19 cell line cultured directly on the glass-coated materials for 10 days, shown as phase contrast images. (**C**) Percentage of RPE cell viability investigated by MTT assay after 10 days of culture with each material (*n* = 3). The 70% dotted line represents the minimum viability threshold of a safe material according to ISO standards. (**D**) Representative flow cytometry plots from each material after staining with the apoptotic marker annexin V and the necrotic marker propidium iodine (PI). (**E**) Mean percentage of the number of viable and non viable cells (apoptotic and necrotic) from 3 independent flow cytometry experiments on RPE cells with direct contact with the materials. *** *p* < 0.001 as compared to viable; # *p* < 0.05 as compared to viable Cr–Au. (**F**,**G**) Immunofluorescence images of RPE cells stained against the filament actin marker Phalloidin, the RPE cell marker RPE65, the proliferation marker Ki67 and the nuclei marker Hoechst. (**H**) Mean percentage of Ki-67-positive cells to the total number of cells. Scale bar 100 µm for (**B**,**G**) and 25 µm for (**F**). RPE, retinal pigment epithelium; PI, propidium iodine.

In order to determine at the molecular level whether the treatment with 100% material extract induces apoptosis, cells were cultured with the extract for 48 h and then processed by flow cytometry using the apoptotic marker Annexin-V and the necrotic marker propidium iodine (PI). One representative plot (Annexin-V against PI) for each of the materials tested is shown in Figure 2D. Quantitative data obtained from three independent experiments were analyzed and summarized in Figure 3E. Similar to the MTT assay, more than 90% viability with no statistically significant difference was observed between Y6, D18, PFN-Br extract-treated cells and the Cr–Au control. However, a significant difference in mean percentage of viable and non-viable cells was observed between the 100% PDIN extract-treated group and the control indicating a decrease in viability to 65% and around a 35% increase of necrotic, early and late apoptotic cells. Except for PDIN, these results indicate that the treatment of RPE cells with Cr–Au, D18, Y6 and PFN-Br extracts do not induce cytotoxicity after 48 h in culture, confirming that there is no toxicological hazard leakage from these chemical structures in in vitro physiological conditions. 

### 2.3. Cytotoxicity of the Materials by Direct Contact

In order to evaluate the potential cytotoxic effect of the material itself for an extended period of time, the same number of human RPE cells was seeded directly on the materials coated on the glass coverslip and incubated for 10 days in vitro (Figure 3A). Phase contrast images show comparable cell attachment and confluency of RPE cells in direct contact with Cr–Au, D18, Y6 and PFN-Br (Figure 3B). In addition, the MTT assay on these materials shows no difference of cell viability (Figure 3C). Similar results were obtained by apoptosis analysis, except for PFN-Br where a slight but significant decrease in cell viability was observed (Figure 3D,E). In contrast, the cells cultured on PDIN coated glass poorly attached and proliferated and the few attached cells appear flat and elongated (Figure 3B). In addition, and similarly to the extract results, high cytotoxicity was observed by the MTT assay with lower than 70% viability (Figure 3C). Due to its high cytotoxicity, we considered that PDIN is not suitable as a material for medical devices and excluded it from further analysis. 

In addition to the individual materials, we next performed a direct contact cytotoxicity assay on the materials mixture D18/Y6 and D18/Y6/PFN-Br. The phase contrast images (Figure 3B) show the good attachment and survival of RPE cells on D18/Y6, which was also confirmed by MTT assay and apoptosis analysis with more than 90% cell viability (Figure 3C–E). Surprisingly, D18/Y6/PFN-Br show poor cell attachment and the attached cells appear stretched with long and thin filopodia projections (Figure 3B). The MTT assay confirmed the cell culture observation and indicate a very low viability of 50%. To verify if the extracts from the materials mixture have also an impact on cell survival, we analyzed the cytotoxicity and apoptosis of D18/Y6 and D18/Y6/PFN-Br extracts after 48 h as well as 10 days of culture with RPE cells. After 48 h, the viability for both mixtures was above 80% (Supplementary Figure S3A–C). However, after 10 days, the D18/Y6/PFN-Br mixture extract shows a significant reduction in the percentage of viable cells as compared to the control (Supplementary Figure S2D). Due to its poor direct and indirect contact cytotoxicity, we considered D18/Y6/PFN-Br mixture an unsafe material for medical devices.

To further the evaluate cell morphology of the so far non-cytotoxic materials, D18, Y6 and D18/Y6, RPE cells cultured for 10 days on these glass-coated materials were fixed and assessed by immunofluorescence. Cells were stained with Hoechst for nuclei, RPE65 for RPE cells and Phalloidin to stain F-actin (Figure 3F). The fluorescence images indicate typical RPE cell morphology as compared to controls with no apparent nuclei or cytoskeleton dysregulation. In addition, to confirm normal cell proliferation, RPE cells were grown directly on the materials to reach 50–70% confluency, then the expression of the proliferation marker Ki-67 was evaluated. One representative fluorescence image obtained for Cr–Au, D18, Y6 and D18/Y6 is shown in Figure 3G. The percentage of Ki-67-positive cells to the total number of cells reveals no significant difference between D18, Y6 and D18/Y6 as compared to the Cr–Au control (Figure 3H). The overall results indicate that D18 and Y6 alone and D18/Y6 combination are not cytotoxic when confronted in vitro with human RPE cells. 

### 2.4. Functional Assessment of Retinal Explant

**Figure 4:**
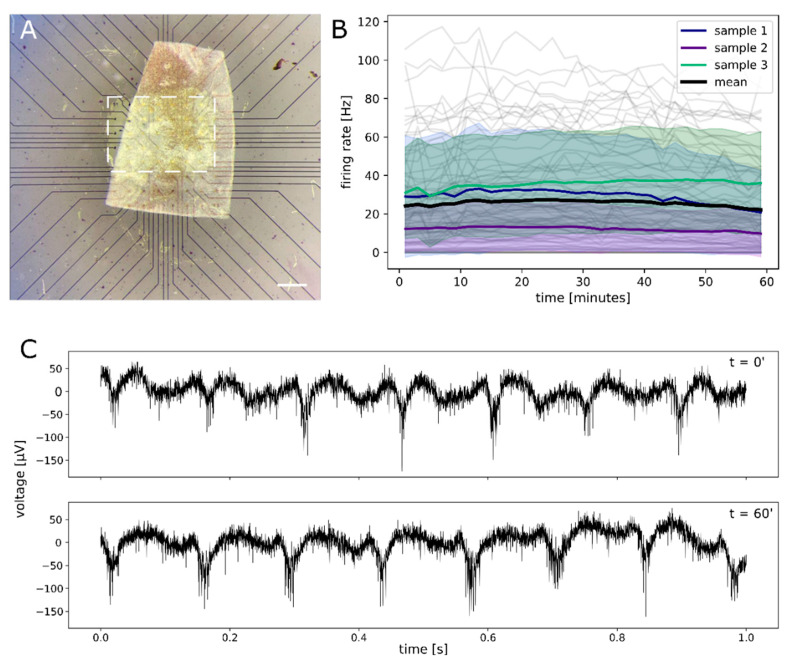
Functional assessment of mouse ex-vivo retina by electrophysiology via microelectrode array (MEA) recording. (**A**) Ex vivo retina sample placed on a glass MEA coated with D18/Y6. The white dashed rectangle indicated the window of the exposed MEA surface to ensure contact between the tissue and the electrodes to enable recording. Scale bar: 400 µm. (**B**) Firing rate during a continuous 1 h recording. The plot shows the average firing rate (FR) values for retinal ganglion cells in 3 different retina samples and the average of the 3 samples (black line—mean). Small gray line indicates FRs on single electrodes, colored areas indicate the standard deviation of the mean FR. (**C**) Characteristic photoreceptor-degenerated retina rhythmic activity (retinal waves) recorded from the same electrode at the start (t = 0′) and the end (t = 60′) of the one-hour continuous recording.

In order to evaluate if D18/Y6 has any effect on the functionality of ex-vivo retina, it was interfaced to photoreceptor-degenerated intact retinal tissue, dissected from adult rd10 mice. The ex vivo retina was interfaced and electrophysiologically recorded via MEAs coated with a D18/Y6 mixture (Figure 4A). Right above the electrodes, D18/Y6 was removed to allow for a direct interfacing. We assessed the possible effects of the photoactive material on the retinal ganglion cells (RGCs) activity by continuously monitoring the spontaneous RGCs firing for 1 h (Figure 4B). The average firing rate (FR, number of action potentials within one second) remained constant for the entire experiment duration indicating no effect of the material on the cells’ activity. Within each of the three experiments, the average firing rate (FR) was calculated based on 27, 32 and 33 electrode data samples, respectively, updated every 2 min. A paired *t*-test performed on the data at the beginning of the experiment and at the end indicated no significant difference in population means (*p* > 0.05) within each of the experiments. The average FR values (29 +/− 5 Hz, 12 +/−2 Hz, 36 +/− 5 Hz for the three retinal samples) are in the same range as reported previously for adult rd10 photoreceptor-degenerated retinae (20 +/− 2 Hz [32] ≈ 7 +/− 3 Hz for 20 weeks old mice [33]) recorded with the same technique, thus suggesting a normal physiological behavior of the tissue. Finally, we evaluated the RGC spontaneous activity to detect rhythmic activity (Figure 4C). Rhythmic activity is a well-known phenomenon in photoreceptor-degenerated retinas [34,35,36]. It was possible to detect the retinal oscillations during the full length of the recording without any change in the activity from the start to the end of the experiment (Figure 4C). The presence of rhythmic waves is an additional indicator of a physiological behavior of the tissue that is not affected by the contact with the D18/Y6 layer.

## 3. Discussion

An in vitro cytotoxicity assessment of medical devices is a first important step required for any device intended to be implanted into a human tissue. The retina is one of the most sensitive tissues in the human body and an adequate evaluation of the materials characterizing a retinal prosthesis is of high importance to enhance its performance and avoid eye inflammation. Here, we assessed the in vitro cytotoxicity of several polymers, D18, Y6, PFN-Br and PDIN, as potential candidates for a retinal prosthesis. These materials were never tested for their cytotoxicity on human retinal cells. Therefore, following ISO standards [28], we assessed in vitro cytotoxicity, cell attachment and proliferation as well as cell morphology and apoptosis of the human RPE cell line cultured indirectly or directly with the materials alone and in mixture. Moreover, electrophysiological recordings were assessed on an ex-vivo mouse retinal explant.

The selection of D18 and Y6 was based on our and previous studies showing their high photovoltaic performance in solar cell devices [27,30]. When we analyzed their absorption spectrum, the polymer D18 as a wide bandgap donor shows strong absorption in the visible light spectrum, whereas the Y6 non-fullerene acceptor has a complementary narrow bandgap, enabling light gathering in the near infrared region. Their combined absorption covers the absorbance spectrum of the human retina, which make them excellent candidates for retinal photovoltaic implant. For the fabrication of high-performance solar cells, OPVs are usually coupled with other molecules such as PDIN and PFN-Br [27,37], so we decided to include them also in this study as additional potential materials for the organic retinal prosthesis.

We first assessed the cytotoxicity on the materials’ extract with an ISO standards indirect cytotoxicity test. This test evaluates the potential hazards that could leach from the materials when exposed to physiological conditions. This assessment shows that media exposed to the materials D18, Y6, PFN-Br, D18/Y6/PFN-Br and D18/Y6 for 48 h were safe for human RPE cells at any of the concentrations tested. In order to detect traces of apoptotic and necrotic cells at early stages, we performed flow cytometry using Annexin V/PI staining. Similar results were obtained for these materials, showing a high percentage of viable cells as compared to the control. In addition, phase contrast images and immunostaining show good cell attachment with no apparent morphological changes. For PDIN, however, cytotoxicity effects were observed on its extract with RPE cell viability plunging below 70%, which is the minimum threshold required for a safe material. A low number of viable cells was also confirmed by flow cytometry, and different morphology was observed when compared to controls. N,N′-Bis [3-(dimethylamino)propyl]perylene-3,4,9,10-tetracarboxylic diimide, known as PDIN, is a traditional cathode interface material (CIM) and can be used as an electron-transporting layer (ETL) material to effectively modify and enhance surface morphology [38,39]. PDIN’s cytotoxicity on RPE cells was not previously assessed, but its derivative perylene was proven cytotoxic for HeLa cells [40] and the human bronchial cell line NL20 [41]. In concordance with these reports, we here provided additional evidence of the cytotoxicity of PDIN on human RPE cells. 

The second cytotoxicity assessment was performed by direct contact between the materials and the RPE cells. This is a crucial evaluation indicating the capacity of retinal cells to make close contact with the materials of the retinal prosthesis after implantation. Similar to what we obtained by indirect contact, D18, Y6 and D18/Y6 show no significant difference between them and the control when cytotoxicity, apoptosis, morphology and proliferation were analyzed. A more drastic cytotoxicity effect was again observed for PDIN, validating the results of its extract. However, PFN-Br and D18/Y6/PFN-Br materials show different cytotoxicity outcomes by direct contact. By the MTT assay, the PFN-Br material alone did not show cytotoxicity but the apoptosis analysis indicates a significant increase in the number of apoptotic cells after 10 days of direct contact with the materials. When PFN-Br was combined with D18 and Y6, a drastic cytotoxicity effect appeared after 10 days of direct and indirect contact with the RPE cells. This cytotoxicity of PFN-Br by direct and indirect contact indicates that this material is not suitable to be in close contact with retinal cells. 

Because of its hydrophobic backbone and hydrophilic side chains, PFN-Br, or (Poly[(9,9-bis(3′-(N,N-dimethylamino)propyl)-2,7-fluorene)-alt-2,7-(9,9–dioctylfluorene)]dibromide), is a conjugated polyelectrolyte used as an ETL material in organic electronic devices, to improve the interfacial properties such as extraction efficiency [37,42]. The PFN-Br interfacial layer embedded in organic photovoltaic devices gives an overall enhanced open-circuit voltage, short-circuit current density and fill factor, thus improving the device’s performance. The cytotoxicity of this polymer has never been previously assessed in any study; here is the first proof of PFN-Br cytotoxicity when combined with D18 and Y6, in direct and indirect contact for 10 days with human RPE cells. The reason for this low cell survival might be due to the physical interaction between the cells and the surface of the material coated together with D18 and Y6. Previous studies have shown that the nature of the polymer surface, the different surface shapes and the area interface between the electronic organic material and living cells will have important consequences for cell function, attachment and survival [43,44]. Further analysis is still needed to investigate the chemical nature of the surface and the reason for the low cell survival. It is also important to note that the coating procedure of the organic materials on the glass has a critical impact on the RPE cell attachment and survival. To generate high-quality thin films for solar cells, the organic materials are usually processed using either chlorinated solvents such as chlorobenzene and chloroform (for D18 and Y6, respectively) or conventional alcohol solvents such as methanol (for PDIN or PFN-Br) [45,46]. These solvents are highly toxic for biological processes. During our initial cytotoxicity experiments, the RPE cells failed to attach to the glass coated with the organic materials and we encountered massive cell death (Supplementary Figure S4). We then realized that the processing solvents used to coat the organic materials are the reason for this phenomenon. We therefore optimized the coating procedure by using vacuum coating to avoid alcohol solvents, or to imbed the coated glass in water for 24 h to dissolve the chlorinated solvents. Nevertheless, it is worth mentioning that we vacuum-sublimed PFN-Br and PDIN but the cytotoxic effects over the RPE cell line were not different than the ones recorded for the solution-processed materials. In addition, we did not see any difference in neuronal activity on ex-vivo mouse retinas between photocapacitive couples that were immersed in deionized water for removal of the chloroform and those that were used immediately after their deposition and subsequent drying. The negative effect of not removing the solvent was observed on the survival and proliferation of the human RPE cell culture.

All the in vitro cytotoxicity experiments performed on the human RPE cells revealed that D18, Y6, and D18/Y6 are safe organic semiconductor materials. In addition, we coated MEAs with the D18/Y6 and measured a constant spontaneous firing rate (number of RGC action potentials/second) over one hour in the ex vivo retina dissected from rd10 mouse, an animal model of retinal dystrophy. Prior to the recording, the retina rests for at least 30 min on D18/Y6 in carbonated AMES’ medium. This experiment suggested even further the safety of the materials and that D18/Y6 has no impact on the function of the retina, at least within the conditions of the experiment. These initial successful tests will be followed by in vivo biocompatibility tests, as recently reported for conjugated polymer-based retinal prostheses [47]. In addition, further studies will be necessary to carefully assess, for prolonged exposure in biological conditions, the functionality and reliability of the photocapacitive couples upon illumination and its use as a stimulating device with the interfaced retina.

D18, or Poly[(2,6-(4,8-bis(5-(2-ethylhexyl-3-fluoro)thiophen-2-yl)-benzo[1,2-b:4,5-b′]dithiophene))-alt-5,5′-(5,8-bis(4-(2-butyloctyl)thiophen-2-yl)dithieno[3′,2′:3,4;2′′,3′′:5,6]benzo[1,2-c][1,2,5]thiadiazole)], also referred to as PCE18, is a narrow bandgap copolymer with a backbone-alternating electron-donating benzodithiophene (BDT) and electron-accepting fused-ring dithienobenzothiadiazole (DTBT) units. With a larger molecular plane structure and higher degree of conjugation from the fused ring DTBT, D18 has a high hole mobility of 1.59 × 10^−3^ cm2 V^−1^ s^−1^ [30,48]. Y6, or 2,2′-((2Z,2′Z)-((12,13-bis(2-ethylhexyl)-3,9-diundecyl-12,13-dihydro-[1,2,5]thiadiazolo[3,4-e]thieno [2′′,3′′:4′,5’]thieno[2′,3′:4,5]pyrrolo[3,2-g]thieno[2′,3′:4,5]thieno[3,2-b]indole-2,10-diyl)bis(methanylylidene))bis(5,6-difluoro-3-oxo-2,3-dihydro-1H-indene-2,1-diylidene))dimalononitrile, is a popular non-fullerene acceptor (NFA) molecule. The development of new NFA molecules such as Y6 and their use in OPV solar cells has led to rapid improvements in device power conversion efficiencies. Using Y6, significant improvements in solar cell performance have been achieved. Y6 is a highly conjugated electron deficient organic semiconductor with an A-DAD-A structure. The Y6 molecule is composed of a fused thienothienopyrrolo–thienothienoindole (TTP-TTI) core base and 2-(5,6-difluoro-3-oxo-2,3-dihydro-1H-inden-1-ylidene)malononitrile end units, which are thought to enhance optical absorption. In fact, Y6 and its polymer blends have the potential to absorb light across the entire visible and near infra-red spectrum [49,50,51,52]. In addition, a device efficiency of 18.22% and certified efficiency of 17.6% have been achieved using D18 as the electron donor and Y6 as an acceptor in a single-junction non-fullerene polymer solar cell [30].

Taken altogether, the different in vitro cytotoxicity tests performed in this study has provided the first evidence for the safety of D18, Y6 and D18/Y6 organic semiconductors on human RPE cells and mouse retinas. We also confirmed the cytotoxicity of PDIN, as well as showed for the first time that D18/Y6/PFN-Br is not suitable for human RPE cell survival. However, it is important to note that these in vitro results should be complemented with in vivo biocompatibility tests to guarantee the safety and efficacy of these materials for long-term retina implantation.

## 4. Materials and Methods

### 4.1. Coating of the Organic Materials

OPV materials (D18 and Y6), as well as ETL materials (PFN-Br and PDIN) were purchased from 1-Material Inc. The OPV layers were processed from solution using the spincoating technique. For pristine D18 and Y6 films, a 5 mg/mL solution in chlorobenzene and a 15 mg/mL in chloroform was used, respectively. Active layer solutions of the donor:acceptor blend D18:Y6 were prepared following the procedure described in [27], where a solution with a D/A weight ratio of 1:1.6 and a concentration of 9 mg/mL in chloroform was used. For pristine and blend films, the spincoating was performed at 2500 rpm for 30 s. The deposited film was immersed in deionized water overnight and then dried extensively by leaving it in a vacuum of 10^−6^ torr for 12 h. PDIN was dissolved in a mix of methanol and glacial acetic acid with a volume ratio of 1000:3 with a concentration of 2 mg/mL. A spinspeed of 5000 rpm for 30 s was used to obtain thin PDIN layers with a thickness below 10 nm. PFN-Br was dissolved in methanol with a concentration of 0.5 mg/mL and was spincoated at 2000 rpm for 30 s. The thickness of coated materials was 90 to 100 nm depending on the material and the coating procedure used, which is representative of the thickness that will be used in the real device.

### 4.2. Absorbance Measurement

A double-beam UV-vis-NIR spectrometer form PerkinElmer (Lambda 1050) was used to measure the optical transmission (T) of D18 and Y6 thin films on glass. The optical absorbance (A) was calculated using the negative decadic logarithm of the transmission (A = −log_10_(T)).

### 4.3. Human RPE Cell Culture

Human retinal epithelium ARPE-19 cells from ATCC (#CRL2302, LGC Standards Gmbh, 46,285 Wesel, Germany) were cultured in complete Dulbecco′s Modified Eagle Medium F12 (1132007-DMEM/F12, Gibco^®^, Thermo Fisher Scientific, Dreieich, Germany,) medium containing 10% fetal bovine serum (FBS) (10082147-Gibco^®^, Thermo Fisher Scientific, Dreieich, Germany) and 1% penicillin/streptomycin (100 unit penicillin/100 μg streptomycin per milliliter) (Invitrogen ^®^, Thermo Fisher Scientific, Dreieich, Germany). Cell cultures were maintained in T75 tissue culture flasks or 6/12-well tissue culture plates (StarLab, CytoOne^®^, Hamburg, Germany) in standard culture conditions of 37 °C and 5% CO_2_, changing the media every 2 days. The cells were harvested by trypsinization using 0.05% Trypsin–EDTA solution (Gibco^®^, Thermo Fisher Scientific, Dreieich, Germany) at 80–90% culture confluence and further sub-cultivated into culture flasks. The ARPE-19 (passage 23 to 27) cells were cultured either directly on the sterilized organic-material-coated coverslips or in extract obtained after 24 h incubation of the sterilized organic-material-coated coverslips in culture media with a seeding density of 3 × 10^4^ cells per well of a 12-well plate. Sterilization of the organic-material-coated coverslips was performed at 180 °C for 8 h. 

### 4.4. MTT Cytotoxicity Assay

To perform MTT cytotoxicity assays on the extract, the organic materials coated on glass coverslips were incubated in culture medium for 24 h at 37 °C. Following ratios of extraction media to test samples defined by ISO10993-12 [31], we used 1 mL of culture medium for extracting the samples with area of 22 mm × 22 mm. The samples’ extract were then collected and diluted with culture medium to obtain samples with 0 (control), 25, 50, 75 and 100% concentrations. 0.05 Triton-X-100 was used as a positive cell death control. ARPE-19 cells were seeded to a 96-well plate at seeding density of 1 × 10^4^ cells per well. After incubation for 24 h, the culture medium was removed and replaced with extract samples, and incubated for 2 and 10 days. The number of living cells after incubation was quantified by the MTT assay. Three replicates were included in each group. At the desired assay time, 120 μL of diluted MTT reagent (T8787, Merck KGaA, Darmstadt, Germany) (5 mg/mL) in MEM medium (11095080-Gibco^®^, Thermo Fisher Scientific, Dreieich, Germany) was added to the wells and incubated at 37 °C for 2 h. The MTT solution was then discarded, and 100 μL of isopropanol (I9516, Merck KGaA, Darmstadt, Germany) was added. The plates were placed on a shaker to solubilize the formations of purple crystal formazan. Cell viability was determined by measuring the optical density at 560 nm using a microplate reader (GloMax^®^ Explorer, Promega, Madison, WI, USA). The intensity of the resulting optical absorbance was proportional to the number of living cells. 

For samples from direct contact, ARPE-19 cells were grown in 12-well plates on the coated glass coverslips and incubated for 10 days. The number of living cells after incubation was quantified by the MTT assay. Three replicates were included in each group. A Cr–Au coated coverslip was used as negative control. At the desired assay time, 500 μL of diluted MTT reagent (T8787- Merck KGaA, Darmstadt, Germany) (5 mg/mL) in MEM medium (11095080-Gibco^®^, Thermo Fisher Scientific, Dreieich, Germany) was added to the wells and incubated at 37 °C for 2 h. The MTT solution was then discarded, and 1 mL of isopropanol (I9516, Merck KGaA, Darmstadt, Germany) was added. The plates were placed on a shaker to solubilize the formations of purple crystal formazan. Cell viability was determined by measuring the optical density at 560 nm using a microplate reader.

### 4.5. Apoptosis Analysis by Flow Cytometry

For apoptosis analysis, an Apoptosis Detection Kit was employed (4830-01-K; R&D Systems, Minneapolis, MN, USA). ARPE-19 cells were treated with Trypsin (15400054-Gibco^®^ Thermo Fisher Scientific, Dreieich, Germany) before being collected by centrifugation and washed twice in PBS (BE02-017F-Lonza, Walkersville, MD, USA). The suspension of cells was stained with a 1:100 dilution of the TACS Annexin V-FITC and a 1:10 dilution of Propidium lodide (PI) for 15 min in the dark; resuspended in the provided binding buffer and immediately processed by flow cytometry. Cr–Au was used as the negative control. A total of 10,000 events per sample of ARPE-19 cells were acquired with the flow cytometer (CytoFlex V2, Beckman Coulter, Inc., Brea, CA, USA). All the data were analyzed by CytExpert V.7.6 (Tree Star, Inc., Ashland, OR, USA) and Kaluza V 2.1 (Beckman Coulter, Inc., Brea, CA, USA). The gating strategy is showed in Supplementary Figure S2E.

### 4.6. Immunofluorescence

RPE cells plated on glass-coated coverslips were washed in PBS (BE02-017F-Lonza, Walkersville, MD, USA) and fixed in 4% PFA (158127-Merck KGaA, Darmstadt, Deutchland) for 10 min. After washing, the cells were permeabilized with 0.3% Triton-X-100 (T8787- Merck KGaA, Darmstadt, Germany) in TBS 0.1 M for 15 min and then incubated for 1 h in the blocking solution containing 0.025% Triton X-100 and 10% fetal bovine serum (10082147-Gibco^®^ Thermo Fisher Scientific, Dreieich, Germany) in TBS 0.1 M. Subsequently, they were incubated overnight at 4 °C with the primary antibodies diluted in the blocking solution containing 0.025% Triton X-100 and 5% fetal bovine serum in TBS 0.1 M. On the next day, cells were washed in 0.1 M PBS/0.025% Triton X-100 (TBST) and incubated with the corresponding fluorescent secondary antibodies (Invitrogen^®^, Thermo Fisher Scientific, Dreieich, Germany). After washing with TBST, the cell nuclei were stained with Hoechst 33,342 (H3570, Invitrogen^®^, Thermo Fisher Scientific, Dreieich, Germany) for 5 min and then washed again with distilled water. Finally, the cells on coverslips were mounted onto glass slides using Aqua- Poly/Mount (18606, Polyscience^®^, Hirschberg an der Bergstrasse, Germany). The following antibodies have been employed: RPE65 (1:50, sc-390787, Santa Cruz Biotechnology, Inc., Heidelberg, Germany), Ki67 (1:100, 27309-1-AP, Proteintech, Manchester, United Kingdom), Phalloidin (1:200, A30106, Invitrogen^®^, Dreieich, Germany). The stained RPE cells were examined under the Zeiss LSM 780 inverted confocal microscope (Zeiss^®^, Jena, Germany). Further image analysis has been performed through the ImageJ software, version 1.53c (Wayne Rasband, National Institutes of Health, Bethesda, MD, USA). For Ki67 counting, 10 different visual fields from each slide (2 slides for each material tested) were taken with 10× magnification and semi-automatic counting of Ki-67-positive cells was performed using ImageJ software. The Ki-67 index was determined from the Ki-67-positive cells and the total number of cells in one visual field based on DAPI staining. 

### 4.7. Electrophysiological Recordings

Retina electrophysiological data were collected from three different retinal samples isolated from 2 adult photoreceptor-degenerated mice (rd10) (130 and 165 days old). The experimental procedures for preparation of the ex vivo retina were reported and approved by the Center for Biomedical Research, Medical University Vienna, Austria. After enucleation of the eye a portion of the retina, circa 1.5 × 2 mm^2^, was isolated and placed on the coated microelectrode array (MEA) with retina ganglion cells in close contact with the recording gold microelectrodes (Figure 3A). During the preparation, the retina was maintained in carbonated (95% O_2_, 5% CO_2_) Ames’ medium (Ames A 1420, Sigma Aldrich + NaHCO_3_) at room temperature. The experimental protocol consisted of 30 min of dark adaptation of the retina after its placing on the MEA, followed by one hour of continuous recording. The tissue was kept in the dark for all the duration of the recording.

Recordings were performed using a conventional glass MEA (59 recording electrodes, 30 µm diameter, 200 µm spacing, Multi Channel Systems MCS GmbH) coated with D18/Y6. A window in the coating was opened manually to guarantee direct contact between the retina and the electrodes and allow recording (Figure 3A). 

Analysis of RGC’s spiking activity was performed via single channel threshold crossing [53] using a threshold limit of 5.5 times the standard deviation of the high-pass (200 Hz) filtered extracellular voltage. To evaluate the RGC spiking activity during the one hour experiment, RGC firing rates (spikes/s) were calculated within 2 min intervals (Figure 4B) and represented as a continuous line. Only electrodes which detected at least 60 spikes/h are included in the analysis. The mean FR values are calculated over the 1 h-long recording. Deviations of the mean FR are presented as standard error of the mean.

### 4.8. Stastical Analysis

For statistical analysis, a two-way analysis of variance ANOVA followed by the Neuman–Keuls multiple comparison test was used for the comparison of the mean values of the flow cytometry data. For Ki67 counting, a one-way ANOVA was performed. A paired *t*-test was assessed for the MEA experiments. In the figures, * *p* < 0.05, ** *p* < 0.01 and *** *p* < 0.001. Error bars represent the standard error of the mean (SEM) in all bar graphs. Three independent biological replicates were included in all analyses.

## 5. Conclusions

The field of electronic retinal prosthesis and the emergence of organic semiconductors represent an encouraging therapeutic strategy to restore vision to retinal dystrophy patients. Different organic semiconducting polymers have been investigated and tested for their performance and efficacy in retinal stimulation, but their cytotoxicity testing has not been adequately investigated. Therefore, in this study, a comprehensive and ISO standard in vitro retinal cytotoxicity investigation was performed on novel organic materials, namely D18, Y6, PFN-Br, PDIN and their photocapacitive couple configurations D18/Y6, D18/Y6/PFN-Br and D18/Y6/PDIN. In contrast to PDIN and PFN-Br, we found that D18, Y6 and D18/Y6 are fully safe in vitro on retinal cells with regards to cytotoxicity, apoptosis, cell morphology and proliferation, as well as spontaneous electrical firing. Therefore, and due to their proven high performance as solar cells, we conclude the D18/Y6 can be a suitable candidate as a photocapacitive couple for retinal prostheses, which will be the focus of our future investigation.

## Data Availability

Not applicable.

## Supplementary Materials

The following supporting information can be downloaded at: https://www.mdpi.com/article/10.3390/ijms23158666/s1.
