# Supplementary material for: In Vitro Cytotoxicity of D18 and Y6 as Potential Organic Photovoltaic Materials for Retinal Prostheses
Source: Int J Mol Sci. 2022 Aug 4;23(15):8666. doi: 10.3390/ijms23158666 (PMC9369111; doi:10.3390/ijms23158666)
Supplement: Supplementary file 1 [file ijms-23-08666-s001.zip › ijms-1832763-supplementary.pdf]

PDIN

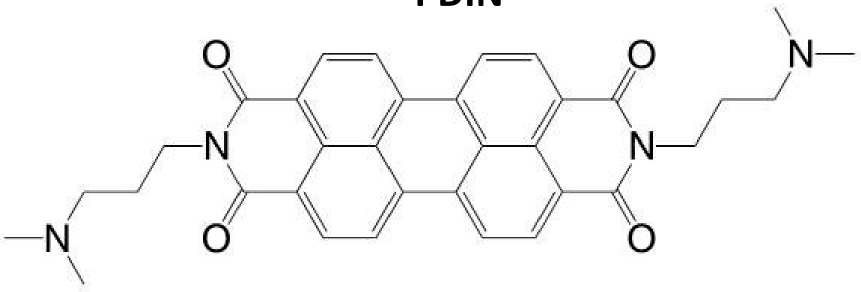

PFN-Br

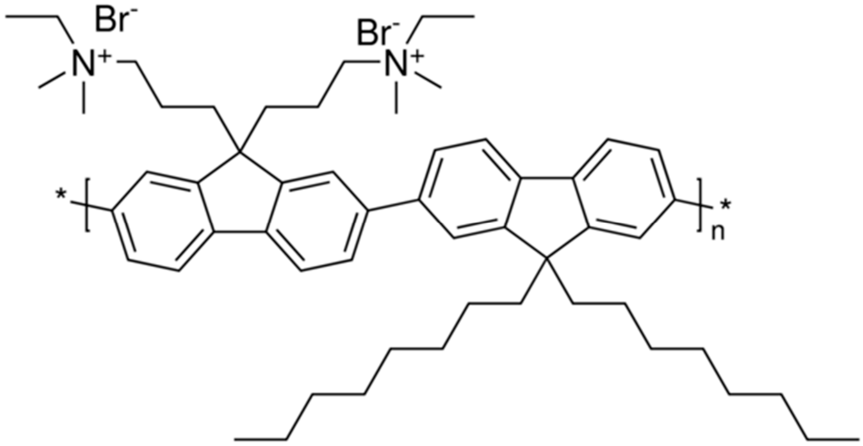

Figure S1. Chemical structures of PDIN and PFN.

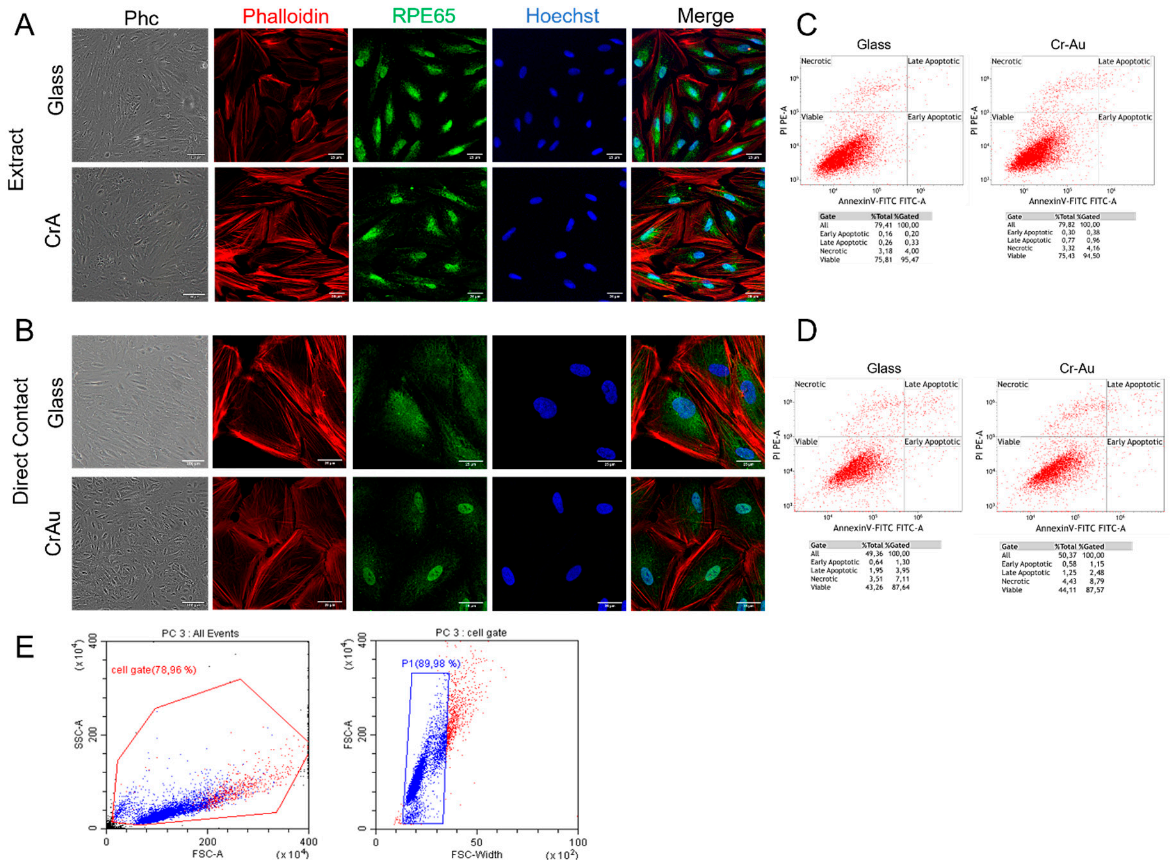

Figure S2. No difference in cell viability between uncoated and Cr-Au coated glass. A) RPE-19 cell line cultured with the glass and Cr-Au extracts for 48 hours, shown as phase contrast and immunofluorescence images stained against the filament actin marker phalloidin , the RPE cell marker RPE65 and the nuclei marker Hoechst. B) RPE-19 cell line cultured directly on uncoated and Cr-Au coated glass for 10 days, shown as phase contrast and immunofluorescence images stained against the filament actin marker phalloidin , the RPE cell marker RPE65 and the nuclei marker Hoechst. C-D) Representative flow cytometry plots from indirect (C) and direct contact (D) between RPE cells, coated and uncoated glass, after staining with the apoptotic marker annexin V and the necrotic marker propidium iodine (PI). The tables underneath each plot represent the percentage of the cells from each quadrant. E) Gating strategy for the flow cytometry experiments. First gating strategy employed was based on the size or granularity of cells. From the forward and side 2D scatter plot, the cell debris were excluded. Second gating strategy relied on excluding the cell doublets or aggregates from measurements. This was achieved with the forward scatter height to width/area ratio where only single cells were appropriately gated. Scale bar 100 µm for the Phc images and 25 µm for the fluorescent images. RPE, retinal pigment epithelium; Phc, phase contrast; PI, propidium iodine.

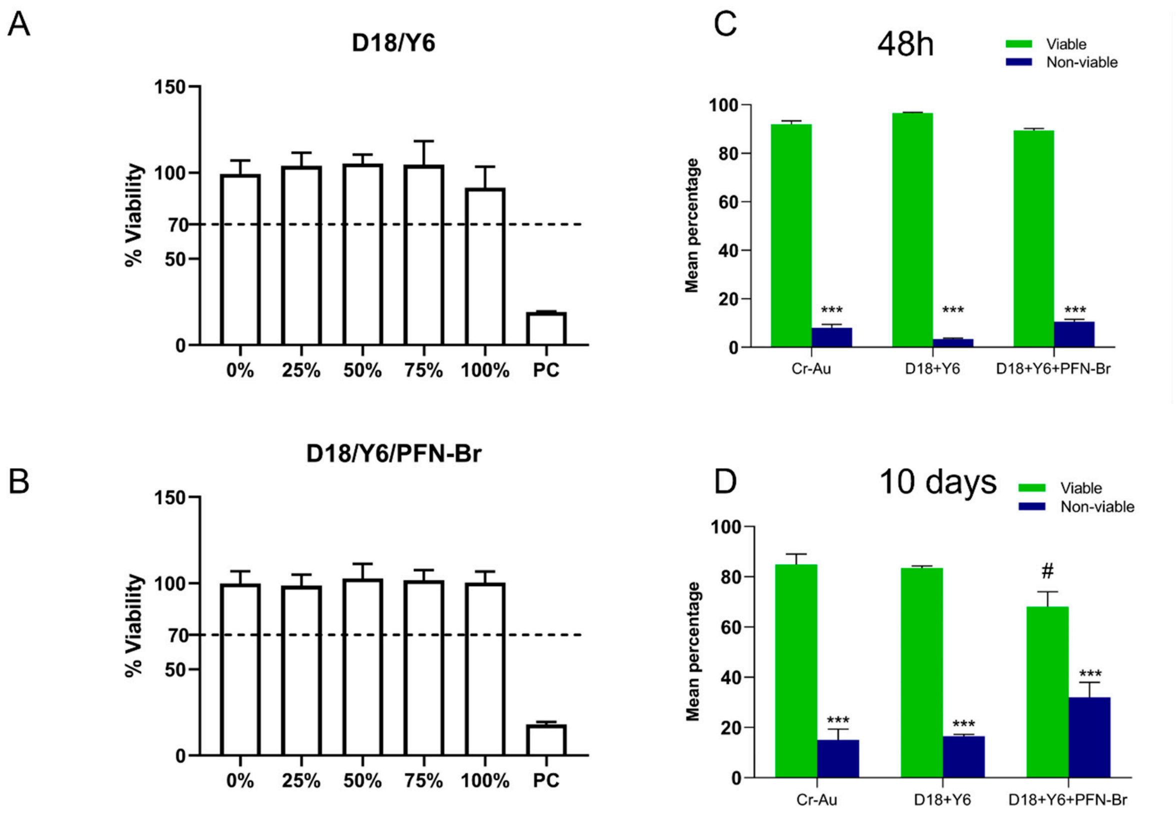

Figure S3. Cytotoxicity of the D18/Y6 and D18/Y6/PFN-Br extracts after 48 hours and 10 days. A, B) Percentage of RPE cell viability investigated by MTT assay after 48 hours of culture with each material extract at different concentrations (n=3). The 70% dotted line represent the minimum viability threshold of a safe material according to ISO standards. C,D) Mean percentage of the number of viable and non viable cells (apoptotic and necrotic) from 3 independent flow cytometry experiments on RPE cells cultured with the extracts for 48 hours (C) or 10 days (D). *** $p<0.001$ as compared to viable; # $p<0.05$ as compared to viable Cr-Au. Phc, phase contrast; PC, positive control.

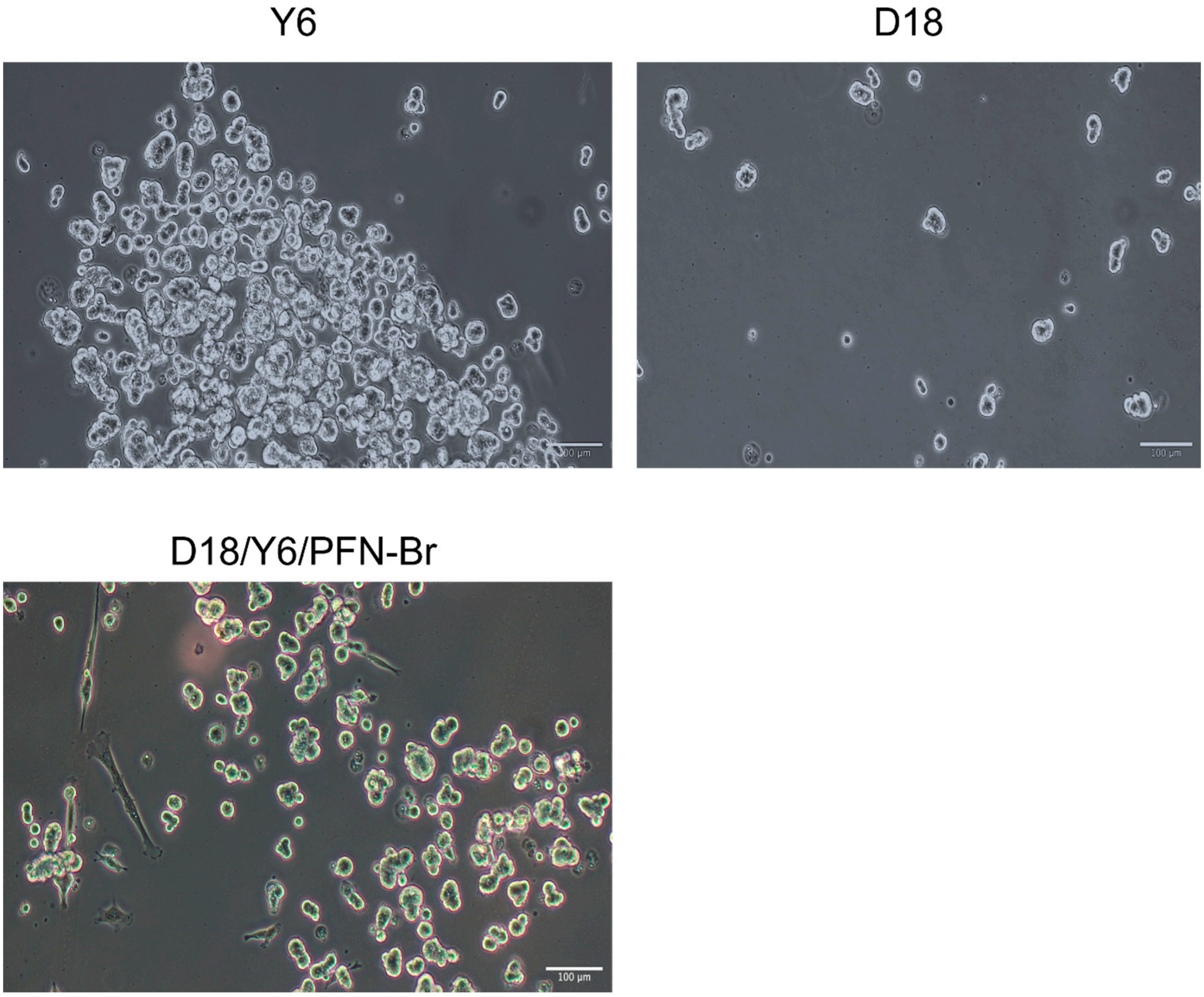

Figure S4. The coating procedure has an impact on cell attachment. Phase contrast images showing no attachment of RPE cells on Y6, D18 and D18/Y6/PFN-Br coated glass when we did not cleared the chlorinated solvents used to dissolve and coat the materials on the glass substrate. Scale bar, 100 µm.
